# Patient-reported outcome after rheumatoid arthritis-related surgery in the lower extremities: A report from the Swedish National Register of Rheuma Surgery (RAKIR)

**DOI:** 10.3109/17453674.2011.645193

**Published:** 2012-04-24

**Authors:** Anna Clara Benoni, Ann Bremander, Anna Nilsdotter

**Affiliations:** ^1^Research and Development Department, Halmstad Central Hospital, Halmstad; ^2^Research and Development Center, Spenshult Hospital for Rheumatic Diseases, Oskarström; ^3^Department of Orthopedics, Clinical Sciences, Lund University, Lund, Sweden

## Abstract

**Background and purpose:**

Although decreasing with the development of effective pharmacological regimes, joint surgery has improved the function and quality of life of patients with rheumatoid arthritis (RA). Few studies have assessed patient-reported outcomes after RA surgery to the lower extremities. Here we report patient-relevant outcome after RA-related surgery based on the first data from the Swedish National Register of Rheuma Surgery (RAKIR).

**Patients and methods:**

258 RA patients (212 women) who had joint surgery performed at the Department of Orthopaedics, Spenshult Hospital between September 2007 and June 2009 were included. Mean age at surgery was 64 (20–86) years. The patients completed the SF-36 and HAQ questionnaires preoperatively and 6 months postoperatively, and 165 patients completed them after 12 months.

**Results:**

Improvement was seen as early as at 6 months. At 12 months, 165 patients (141 women)—including hip (n = 15), knee (n = 27), foot (n = 102), and ankle (n = 21) patients—reported statistically significant improvements from preoperatively to 12 months postoperatively in HAQ (mean change: –0.11) and SF-36 subscales physical function (11), role physical (12), bodily pain (13), social functioning (6.4), and role emotional (9.4). Hip and knee patients reported the greatest improvements.

**Interpretation:**

Orthopedic RA-related surgery of the lower extremities has a strong effect on pain and physical function. Improvement is evident as early as 6 months postoperatively and remains after 12 months.

Rheumatoid arthritis (RA) can have a major effect on health-related quality of life and can lead to reduction in physical function and in mental health (Doyle 2001, Kosinski et al. 2002, Boonen and Severens 2011). Orthopedic surgery has improved the function and quality of life of patients with RA. A decreasing rate of RA-related orthopedic surgery in the Nordic countries has been reported (Eskelinen et al. 2006, Weiss et al. 2006, Kapetanovic et al. 2008, Havelin et al. 2009, Robertsson et al. 2010) and has been explained by the development of more effective pharmacological regimes (da Silva et al. 2003, Kapetanovic et al. 2008, Uhlig et al. 2008, Weiss et al. 2008, Söderlin et al. 2010). However, if medical treatment fails, surgical intervention is needed. Patient-related outcome measures are frequently used to quantify the effectiveness of treatment and the long-term effect of RA on the patient's functioning and well-being (Kosinski et al. 2000, Linde et al. 2008, Uhlig et al. 2008). Some recent studies have evaluated the effect of orthopedic interventions in other patient groups (Nilsdotter et al. 2009, Jansson and Granath 2011). Few studies have assessed patient-relevant outcomes after orthopedic surgery in patients with RA.

The Swedish National Register of Rheuma Surgery (RAKIR) was established in 2007 and has been used at Spenshult Hospital for Rheumatic Diseases. The main aim of RAKIR is to measure how surgical procedures affect pain, function, activity, and quality of life in RA patients, in order to improve the results. Data on patient-reported pain, function, and health-related quality of life of patients undergoing surgery have been registered since September 2007, together with data on complications and revision surgery. The objective of this study was to examine the effect of RA-related surgical interventions in the lower extremities, as measured by the patient-administered questionnaires HAQ and SF-36. This analysis is based on the first data from RAKIR.

## Methods

### Patients

Between September 2007 and June 2009, 516 operations in the lower extremities were performed in 439 patients at Spenshult Hospital for Rheumatic Diseases (Figure 1). Patients undergoing surgery were asked to complete the patient-administered questionnaires SF-36 and HAQ preoperatively at the ward. The same questionnaires were sent to the patients by mail 6 months and 12 months postoperatively without any reminders. The procedures were all RA-related joint surgery (Table 1). Operation codes were divided into 4 groups according to the anatomical region: hip, knee, foot, and ankle. Preoperative data were available for 261 patients. 28 patients underwent surgery twice or more, and completed the preoperative questionnaires at least once during the study period, but only data referring to the first intervention were analyzed. 3 patients were excluded either due to surgery of the upper extremity in the same session (n = 1) or due to operations unrelated to RA (n = 2). Data for the remaining 258 patients (212 women) were analyzed: 26 hip patients, 39 knee patients, 162 foot patients, and 31 ankle patients. Mean age at surgery was 64 (20–86) years. 98 patients had had one or more previous intervention performed, including hand/wrist (n = 49), elbow (n = 16), shoulder (n = 7), back or neck (n = 9), hip (n = 21), knee (n = 30), and foot (n = 60) patients. The individual change in SF-36 and HAQ scores was calculated as the difference between scores preoperatively and at 12 months postoperatively. Peroperative complications were reported in 7 patients (hip, n = 2; foot, n = 2; ankle, n = 2; knee, n = 1). Postoperative complications were reported in 3 patients.

**Figure:**
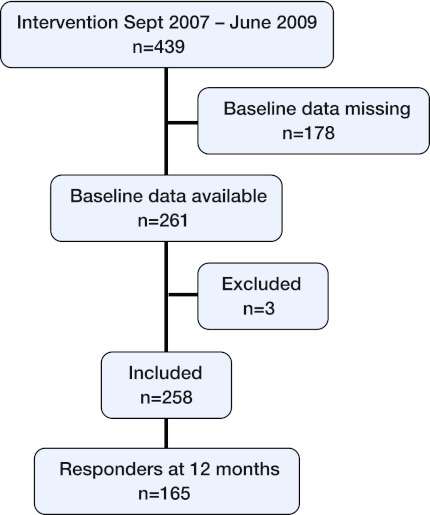
Flow chart of patients.

**Table 1.** Orthopedic interventions

Intervention	n (%)
Forefoot arthrodesis	77 (29)
Stainsby	31 (11)
Hindfoot arthrodesis	37 (13)
Ankle arthroplasty	13 (5)
Ankle arthrodesis	17 (6)
THR	26 (9)
TKR	34 (12)
Forefoot osteotomy	18 (7)
Phalang resection	9 (3)
Hindfoot osteotomy	2 (1)
Knee arthroscopy	2 (1)
Others	9 (3)

### Questionnaires

The Medical Outcome Study Short Form-36 (SF-36) (Ware and Sherbourne 1992) includes one multi-item scale that assesses 8 dimensions of health: physical functioning (PF), social functioning (SF), role limitations because of physical health problems (RP), bodily pain (BP), general mental health (psychological distress and well-being; MH), limitations in usual role activities because of emotional problems (RE), vitality (energy and fatigue; VT), and general health perceptions (GH). Each subscale is scored from 0 to 100, with 0 indicating extreme problems and 100 indicating no problems. The SF-36 is a widely used generic measure of health status and has been validated for Swedish conditions (Sullivan et al. 1993).

The Stanford Health Assessment Questionnaire (HAQ) disability index (Fries et al. 1980) is a disease-specific questionnaire containing 20 items and assesses the degree of difficulty in performing activities of daily living during the previous week. The following response categories are available for each question: without any difficulty (score 0), with some difficulty (score 1), with much difficulty (score 2), or unable to do (score 3). The activities are grouped into 8 dimensions: dressing and grooming, rising, eating, walking, hygiene, reach, grip, and other activities. The HAQ is widely used internationally in patients with RA—in clinical practice as well as in research. It has been translated and validated for Swedish conditions (Ekdahl et al. 1988).

### Statistics

For comparison of preoperative and follow-up questionnaire data, Friedman's test (comparing baseline data, 6-month data, and 12-month data) and Wilcoxon's signed rank test (comparing pairwise data between 2 different examinations, e.g., baseline data and 12-month data) were used. To quantify mean changes in measured outcomes during follow-up, the paired t-test was used for computation of 95% confidence intervals (CIs). For comparisons between groups, the Mann-Whitney test was used. Continuous outcomes are given as mean (SD) since this is customary in describing SF-36 data. Values of p < 0.05 were considered significant.

Power analysis was performed before the start of the study. For a difference of 10 units in the SF-36 subscales and a difference of 0.3 units in the HAQ, with a power of 80% between preoperative and postoperative data (p < 0.05), we calculated that 35 patients per group would be required.

We carried out sensitivity analysis to address the issue of missing data (loss to follow-up). The sensitivity of the reported statistical results for HAQ and SF-36 data was evaluated by imputing baseline data (for the non-responders). Pre- and postoperative data, including the imputed values, were then compared using Wilcoxon's signed rank test. Statistical analysis was performed with the SPSS software version 17.0.

### Ethics

The ethics regional board of Lund approved the study (no. 521/2007).

## Results

At the 12-month follow-up, responses were available from 165 patients (141 women) with a mean age at surgery of 64 (27–84) years (Table 2). The distribution of surgery was as follows: foot (n = 102), ankle (n = 21), knee (n = 27), and hip (n = 15). 93 patients did not complete the 12-month follow-up questionnaires. There were no statistically significant differences between responders and non-responders regarding age, preoperative HAQ score, or preoperative SF-36 score. Friedman's test showed a difference in mean ranks for HAQ and for SF-36 subscales PF, RP, BP, and SF (Table 3). Patients reported statistically significant improvements in scores from before surgery to 12 months postoperatively in HAQ and in SF-36 subscales PF, RP, BP, SF, and RE. Statistically significant improvements were evident at 6 months (compared to preoperatively) in the same subscales, with the exception of RE. VT improved at 6 months but not at 12 months. When the subgroups were analyzed separately, foot patients reported significant improvement in SF-36 PF, RP, and BP scores at 6 and 12 months relative to preoperative levels (Table 4). Ankle patients had significant improvements in BP and SF compared to preoperatively (Table 5). Ankle patients who underwent total joint replacement (n = 10) had significant improvement in pain, and the improvement in PF was close to significant (p = 0.05) while ankle patients who underwent arthrodesis surgery (n = 11) only had significant improvement in SF. Knee patients (Table 6) had significant improvements in HAQ and in SF-36 PF, RP, BP, VT, SF, and RE at 12 months. Hip patients had significant improvements in HAQ and in SF-36 PF, BP, VT, SF, and RE at 12 months (Table 7).

**Table 2.** Patient characteristics

	Responders	Foot	Ankle	Knee	Hip	Non-responders
n	165	102	21	27	15	93
Women, n (%)	141 (86)	94 (92)	16 (76)	20 (74)	11 (73)	71 (76)
Age in years **^a^**	64 (11)	65 (11)	62 (13)	68 (9)	61 (14)	62 (13)
Preoperative HAQ score **^a^**	1.1 (0.58)	1.0 (0.56)	1.4 (0.56)	1.2 (0.58)	1.3 (0.60)	1.1 (0.60)
Preoperative SF-36 scores **^a, b^**						
PF	39 (23)	44 (23)	28 (17)	35 (22)	28 (22)	41 (23)
RP	29 (39)	34 (41)	19 (32)	27 (37)	15 (31)	29 (36)
BP	37 (21)	40 (21)	34 (22)	36 (17)	25 (26)	36 (21)
GH	52 (21)	51 (21)	51 (19)	51 (20)	59 (22)	52 (22)
VT	47 (25)	51 (25)	38 (19)	48 (21)	33 (25)	45 (24)
SF	69 (26)	73 (26)	56 (25)	69 (23)	60 (31)	68 (25)
RE	58 (45)	64 (44)	51 (44)	42 (49)	60 (44)	62 (42)
MH	73 (19)	76 (19)	63 (22)	71 (18)	79 (21)	74 (19)

**^a^** Values are mean (SD)

**^b^** See Methods for a full explanation of the 8 dimensions of the SF-36.

**Table 3.** HAQ and SF-36 scores before and after surgery (n = 165)

	Baseline	6 months	12 months	Mean change (SD; CI)	p-value (Friedman's test)	p-value
HAQ score, mean (SD)	1.1 (0.58)	0.99 (0.62)	1.0 (0.62)	–0.11 (0.44; –0.18 to 0.041)	< 0.001	0.006
SF-36 scores, mean (SD)						
PF	39 (23)	49 (24)	50 (25)	11 (23; 7.3–14)	< 0.001	< 0.001
RP	29 (39)	44 (41)	41 (42)	12 (41; 5.8–19 )	0.001	< 0.001
BP	37 (21)	52 (24)	50 (24)	13 (24; 9.5–17)	< 0.001	< 0.001
GH	52 (21)	52 (23)	50 (21)	–2.2 (19; –5.0 to 0.70)	0.6	0.2
VT	47 (25)	52 (24)	51 (23)	3.5 (21; 0.24–6.8)	0.1	0.08
SF	69 (26)	77 (25)	75 (26)	6.4 (24; 2.7–10)	< 0.001	0.001
RE	58 (45)	67 (42)	67 (42)	9.4 (48; 1.7–17)	0.2	0.01
MH	73 (19)	77 (20)	74 (20)	0.35 (19; –2.7 to 3.4)	0.2	0.6

**Table 4.** HAQ and SF-36 scores before and after surgery: foot (n = 102)

	Baseline	6 months	12 months	Mean change (SD; CI)	p-value
HAQ score, mean (SD)	1.0 (0.56)	0.97 (0.63)	0.96 (0.59)	–0.036 (0.41; -0.12-0.048)	0.8
SF-36 scores, mean (SD)					
PF	44 (23)	50 (24)	51 (24)	7.4 (22; 3.1–12)	< 0.001
RP	34 (41)	45 (40)	42 (40)	8.4 (40; 0.32–17)	0.03
BP	40 (21)	49 (23)	48 (22)	8.0 (20; 3.9–12)	< 0.001
GH	51 (21)	50 (22)	49 (23)	–2.0 (20; –6.0 to 2.0)	0.4
VT	51 (25)	53 (24)	50 (24)	–1.0 (20; –4.9 to 2.9)	0.5
SF	73 (26)	76 (24)	75 (26)	1.7 (24; –3.0 to 6.5)	0.4
RE	64 (44)	66 (42)	67 (42)	3.0 (48; –6.7 to 13)	0.5
MH	76 (19)	77 (18)	72 (20)	–3.4 (20; –7.4 to 0.59)	0.1

**Table 5.** HAQ and SF-36 scores before and after surgery: ankle (n = 21)

	Baseline	6 months	12 months	Mean change (SD; CI)	p-value
HAQ score, mean (SD)	1.4 (0.56)	1.2 (0.53)	1.4 (0.53)	0.00 (0.41; –0.19 to 0.19)	0.8
SF-36 scores, mean (SD)					
PF	27 (17)	36 (19)	33 (22)	5.1 (21; –4.3 to 15)	0.2
RP	19 (32)	26 (39)	27 (41)	8.3 (41; –10 to 27)	0.4
BP	34 (22)	55 (25)	46 (23)	13 (24; 2.0–23)	0.03
GH	51 (19)	52 (20)	46 (20)	–4.0 (14; –11 to 2.8)	0.2
VT	38 (19)	43 (27)	46 (19)	8.1 (20; –1.3 to 17)	0.2
SF	56 (25)	72 (30)	69 (32)	13 (21; 3.7–23)	0.02
RE	51 (44)	59 (45)	55 (46)	5.0 (56; –21 to 31)	0.7
MH	63 (22)	70 (23)	71 (18)	7.8 (20; –1.8 to 17)	0.1

**Table 6.** HAQ and SF-36 scores before and after surgery: knee (n = 27)

	Baseline	6 months	12 months	Mean change (SD; CI)	p-value
HAQ score, mean (SD)	1.2 (0.58)	1.0 (0.62)	1.0 (0.66)	–0.29 (0.29; –0.41 to –0.17)	< 0.001
SF-36 scores, mean (SD)					
PF	35 (22)	52 (21)	55 (22)	20 (19; 12–27)	< 0.001
RP	27 (37)	50 (45)	51 (46)	26 (47; 6.9–44)	0.01
BP	36 (17)	53 (22)	58 (23)	21 (24; 12–31)	< 0.001
GH	51 (20)	49 (22)	50 (18)	–1.4 (16; –8.0 to 5.1)	0.8
VT	48 (21)	53 (22)	56 (21)	8.5 (18; 1.4–16)	0.02
SF	69 (23)	83 (21)	82 (20)	13 (20; 5.7–21)	0.003
RE	42 (49)	67 (44)	69 (41)	27 (46; 8.3–46)	0.007
MH	71 (18)	73(20)	76 (20)	5.1 (14; –0.32 to 11)	0.06

**Table 7.** HAQ and SF-36 scores before and after surgery: hip (n = 15)

	Baseline	6 months	12 months	Mean change (SD; CI)	p-value
HAQ score, mean (SD)	1.3 (0.60)	0.71 (0.66)	0.87 (0.72)	–0.44 (0.61; –0.78 to –0.11)	0.02
SF-36 scores, mean (SD)					
PF	28 (22)	58 (25)	54 (27)	25 (28; 9.8–41)	0.008
RP	15 (31)	50 (41)	35 (45)	20 (39; –1.7 to 42)	0.07
BP	25 (26)	66 (29)	60 (34)	35 (35; 16–55)	0.006
GH	59 (22)	68 (25)	57 (21)	–2.1 (16; –11 to 6.9)	0.6
VT	33 (25)	62 (24)	54 (25)	20 (26; 4.5–35)	0.03
SF	60 (31)	80 (29)	76 (27)	16 (29; 0.034–32)	0.04
RE	60 (44)	79 (35)	84 (35)	24 (39; 3.0–46)	0.04
MH	79 (21)	87 (19)	86 (18)	7.2 (17; –2.4 to 17)	0.2

When we imputed baseline data at 12 months, the significant p-values originally obtained (for PF, RP, BP, SF, RE, and HAQ) were still significant.

## Discussion

Lower limb function has been shown to deteriorate more than upper limb function over a 10-year period in RA patients (Ringen et al. 2008). However, few studies have assessed patient-relevant outcome after surgery to the lower extremities. Osnes-Ringen et al. (2009) compared surgery in the upper and lower extremities and replacement surgery with non-replacement surgery in 255 patients with inflammatory arthropathies (two-thirds of whom had RA). As might be expected, the largest improvement at 12 months—as measured by the standard response mean (SRM)—was for pain reported from the specific joint that had been operated on. SF-36 subscales PF and BP also showed high SRMs, while SRMs for GH and HAQ were lower. In a study by March et al. (2008) assessing costs and outcomes of total joint replacement surgery for 42 Australian RA patients (31 patients undergoing total knee arthroplasty (TKA) and 11 patients undergoing total hip arthroplasty (THA)), the subjects were followed for 12 months postoperatively. The results were similar to ours, with statistically significant improvements in PF and BP for both knee and hip patients, while neither group improved regarding GH. Hip patients also showed better results in subscales related to other aspects of psychology (VT and MH). Similar results have been found for osteoarthritis patients (Bachmeier et al. 2001) and in RA drug trials (Mease et al. 2008, Wells et al. 2008), where aspects of patient psychology were less affected by pharmacological treatment. It has been proposed that the effect of successful RA treatment may first affect relief of pain, mobility, physical function, and physical role activities—and emotional well-being only later (Kosinski et al. 2002). In a follow-up study of osteoarthritis patients undergoing THA (n = 108) and TKA (n = 86) (Bachmeier et al. 2001), the SF-36 at 12 months was less responsive than the WOMAC, which is a disease-specific questionnaire for patients with osteoarthritis of the lower limb (Bellamy et al. 1988). The relative improvement was most pronounced for physical functioning and physical role functioning. Similarly to our data, the GH subscale after TKA remained unaltered.According to the authors this might be explained by the fact that—even after a successful operation—pain and restrictions in daily life remain, making patients rate their general health as insufficient. Moreover, the chronic course of RA can make evaluation of a specific surgical procedure and its effect on the patient more challenging to interpret (Nelissen 2003), which should be kept in mind when comparing RA surgery and surgery for osteoarthritis. In joint replacement studies including both osteoarthritis patients and RA patients (Espehaug et al. 1998, Bullens et al. 2001), RA patients were more satisfied than osteoarthritis patients after surgery. It was suggested that the moderate correlation between satisfaction and postoperative function could be partly explained by the different expectations of the patients preoperatively (Bullens et al. 2001). As for osteoarthritis patients undergoing knee arthroplasty, outcome of pain and function is best after 12 months (Nilsdotter et al. 2009) but major improvement is evident as early as 6 months postoperatively for RA patients. Improvement in pain has high priority in RA patients (Heiberg et al. 2005) and pain remains the most important indication for RA surgery. In our analysis, for every patient group there was a statistically significant improvement in the SF-36 subscale BP postoperatively compared to preoperatively.

One important limitation of our analysis was the large loss to follow-up. This was a retrospective study based on questionnaire data from a register. The majority of patients only had one hospital admission. Follow-up questionnaires were sent by mail at 6 and 12 months without reminders. No statistically significant differences were found in age, preoperative SF-36 scores, or HAQ score between responders and non-responders. The results were sensitive for imputation of baseline data at 12 months for non-responders. Since data were collected from a register, we could not influence the number of follow-up occasions. Repeated evaluations would have been interesting, as the greatest improvement was seen in the first 6 months. We lacked baseline data for a large number of patients, especially early in the inclusion period. One explanation could be an absence of routines for supplying the patients with questionnaires from the start. Hopefully RAKIR will provide opportunities for further follow-up studies in the future.

In conclusion, our data suggest that orthopedic RA-related surgery of the lower extremities has a strong effect on pain and physical function. Improvement is seen as early as 6 months postoperatively and is still apparent after 12 months.
